# Correction: Highly stable color-tunable organic long-persistent luminescence from a single-component exciplex copolymer for *in vitro* antibacterial

**DOI:** 10.1039/d4sc90185a

**Published:** 2024-09-23

**Authors:** Hui Li, Xiaoye Li, Haoran Su, Shuman Zhang, Cheng Tan, Cheng Chen, Xin Zhang, Jiani Huang, Jie Gu, Huanhuan Li, Gaozhan Xie, Heng Dong, Runfeng Chen, Ye Tao

**Affiliations:** a State Key Laboratory of Organic Electronics and Information Displays, Institute of Advanced Materials (IAM), Nanjing University of Posts & Telecommunications 9 Wenyuan Road Nanjing 210023 China iamhli@njupt.edu.cn iamytao@njupt.edu.cn; b Nanjing Stomatological Hospital, Affiliated Hospital of Medical School, Research Institute of Stomatology, Nanjing University 30 Zhongyang Road Nanjing Jiangsu 210008 China dongheng90@smail.nju.edu.cn; c Songshan Lake Materials Laboratory Dongguan Guangdong 523808 China

## Abstract

Correction for ‘Highly stable color-tunable organic long-persistent luminescence from a single-component exciplex copolymer for *in vitro* antibacterial’ by Hui Li *et al.*, *Chem. Sci.*, 2024, https://doi.org/10.1039/d4sc02839b.

The authors regret that two important related studies (ref. 1 and 2 below) were not cited in the originally published work, and wish to include them to increase the comprehensiveness of the paper.

1 Z. Lin, M. Li, R. Yoshioka, R. Oyama and R. Kabe, Oxygen-Tolerant Near-Infrared Organic Long-Persistent Luminescent Copolymers, *Angew. Chem. Int. Ed.*, 2024, **63**, e202314500.

2 Z. Lin, R. Kabe, N. Nishimura, K. Jinnai and C. Adachi, Organic Long-Persistent Luminescence from a Flexible and Transparent Doped Polymer, *Adv. Mater.*, 2018, **30**, 1803713.

The Royal Society of Chemistry apologises for these errors and any consequent inconvenience to authors and readers.

